# Editorial: Molecular, cellular, and ecological processes of haloarchaea

**DOI:** 10.3389/fmicb.2025.1607148

**Published:** 2025-04-24

**Authors:** Yan Liao, Iain G. Duggin, Maria Ines Gimenez, Zhengshuang Hua

**Affiliations:** ^1^Australian Institute for Microbiology and Infection, University of Technology Sydney, Ultimo, NSW, Australia; ^2^Instituto de Investigaciones Biológicas (IIB-CONICET-UNMDP), Universidad Nacional de Mar del Plata, Mar del Plata, Argentina; ^3^Department of Environmental Science and Engineering, University of Science and Technology of China, Hefei, China

**Keywords:** haloarchaea, molecular, cellular, ecological, interaction

## Introduction

Halophilic archaea (salt-loving archaea), termed haloarchaea, inhabit some of the most hypersaline environments on Earth. As one of the largest lineages within the domain of Archaea, they serve as invaluable models for studying archaeal cell biology due to their relative ease of cultivation, rapid growth kinetics, and an expanding repertoire of genetic tools. Their remarkable adaptations at the molecular, cellular, and ecological level have driven significant advancements in various aspects of archaeal biology, including gene expression and regulation, protein synthesis and adaptation, cell shape and division, and evolutionary processes. Additionally, their unique biochemical and physiological traits have paved the way for diverse biotechnological applications.

This editorial highlights the key contributions from nine recently published manuscripts in the Research Topic “*Molecular, cellular, and ecological processes of haloarchaea*.” These studies explore diverse aspects of haloarchaea, including genetic tool development, genomic adaptations, cellular mechanisms, structural dynamics, and ecological interactions.

## Transcriptional adaptation to environmental conditions

The transcriptional behavior of haloarchaea in controlled laboratory conditions may not fully represent their responses in natural environments. Rosselli et al. investigated the metatranscriptomes of the dominant *Haloquadratum walsbyi* from the solar saltern of Santa Pola (Alicante, Spain), comparing its transcription patterns in natural environments to those observed in laboratory-grown strains. This study reveals significant differences in gene expression, likely due to the adaptation of the cultured strain to homogenous laboratory conditions, whereas natural populations respond dynamically to heterogeneous environmental factors such as nutrient competition, viral attack, and other stressors. This study underscores the limitations of extrapolating laboratory-based gene expression data to natural environments and highlights the complex regulatory strategies employed by *H. walsbyi* to thrive in fluctuating hypersaline ecosystems.

## Virus-host interactions in haloarchaea

One of the major stressors shaping the evolution of haloarchaea in natural environments is virus infection, which can drive genomic changes and influence cellular defense strategies. Mercier et al. presented an in-depth characterization of an archaeal tailed virus (HRTV-DL11) and its host *Halorubrum lacusprofundi*, a model organism for cold adaptation. They investigated the virus life cycle, host range, transcriptional responses to infection, and virus escape mutants. Notably, the researchers discovered that a laboratory-derived strain (*ACAM34-UNSW*) had lost its megaplasmid and ~38% of its secondary chromosome, resulting in the absence of major virus defense systems and making the strain highly susceptible to HRTV-DL1 infection. In contrast, the type strain (*ACAM34_DSMZ*) retained these defense mechanisms, preventing virus replication. Comparative infection studies between these strains enabled the identification of host response specifically activated upon the loss of virus defense mechanisms, broadening our understanding of virus-host interactions in haloarchaea.

## Lipid profiles for potential biomarker discovery

Archaea have a unique lipid composition, characterized by isoprenoid alkyl chains and ether linkages to glycerol-1-phosphate. Yao et al. presented a comprehensive lipidomic study of seven haloarchaeal strains, building an in-house lipid library by using high-performance liquid chromatography coupled with mass spectrometry. They identified a total of 162 lipid features, corresponding to 107 lipids that could be assigned to specific strains. Clustering analyses of both core lipids and total lipid profiles closely aligned with the phylogeny of Halobacteria at the order level. The study also demonstrated how lipidomic features can facilitate the linkage of unknown lipid compounds to phylogeny, refining our understanding of archaeal lipid evolution. This work showcases the power of lipidomics in helping decipher archaeal phylogeny, and offers new avenues for evolutionary studies and biomarker discovery.

## Carbohydrate metabolism

Carbohydrate metabolism is essential for haloarchaeal survival in hypersaline environments. Sorokin et al. described the isolation and characterization of a novel haloarchaeal genus and species *Natronoglomus mannanivorans*, capable of utilizing beta-mannans—plant plant-derived polysaccharides—as a primary carbon source. The researchers enriched the cultures with different forms of beta-mannans and isolated four closely related mannan-degrading strains. Phylogenomic analysis placed these isolates within a new genus in the family *Natrialbaceae*, class *Halobacteri*a. These moderate alkaliphiles exhibited extreme halophilicity and aerobic saccharolytic metabolism, enabling them to utilize not only mannans but also cellulose, xylan, and xyloglucan as carbon sources. This study also highlights the metabolic versatility of halophiles in breaking down complex polysaccharides.

## Advances in protein expression and purification

Efficient expression and purification of extremophilic proteins remain a challenge due to issues such as misfolding and aggregation in conventional hosts like *Escherichia coli*. Karan et al. systematically investigated the impact of various purification tags and their placements on protein expression and purification in *Haloferax voclanii*. Their results demonstrated that an N-terminal 8× His-tag or Strep-tag^®^II significantly improves protein production, purity, and yield in *H. volcanii*. The optimal tag positioning varied depending on the protein, with the C-terminal 8X His-tag or Strep-tag^®^II enhancing mCherry expression, and an N-terminal configuration benefiting halophilic alcohol dehydrogenase. Notably, co-positioning 8× His-tag and Twin-Strep-tag^®^ at the N-terminus led to substantially improved expression across all tested proteins including sfGFP. These findings provide valuable insights into purification tag design, offering a framework for optimizing protein production and purification in haloarchaea.

## Nutrient adaptations

Archaeal survival in nutrient-limited environments relies on their ability to regulate essential elements, such as iron, which is crucial for cellular function and microbial physiology. Sailer et al. investigated the proteomic response of *H. volcanii* to iron starvation using data-independent acquisition mass spectrometry. By comparing cells grown under normal and low-iron conditions, this study revealed that iron starvation severely retarded *H. volcanii* growth and altered the abundance of numerous proteins involved in metal transport and cellular homeostasis. They identified the most affected protein, HVO_A0467, a member of RND family permease, is not essential under standard conditions, and may function as a siderophore exporter. This study provides novel insights into siderophore-based metal acquisition strategies in haloarchaea, offering valuable perspectives on microbial survival in iron-limited environments.

## Spatial organization and motility regulation

Cellular organization plays a pivotal role in sharing the survival and adaptability of haloarchaea. Proteins of the ParA/MinD family of ATPases play critical roles in the spatial organization of diverse cellular structures in both bacteria and archaea. Two manuscripts in this Research Topic investigated the roles of MinD proteins in *Haloferax voclanii*, revealing their influence on the positioning of motility structures near the poles of the rod shaped cells. Brown and Duggin investigated the interplay between MinD proteins and the cytoskeletal protein CetZ1 (an archaea-specific homolog of FtsZ and tubulin). A key finding of this work was the identification of MinD2 as a critical regulator of CetZ1 localization and motility. Deletion of *minD2* or *minD4* disrupted CetZ1′s normal distribution, leading to its mislocalization along the midcell and cell edges while preventing its proper accumulation at the cell poles. Similarly, MinD4 also influenced CetZ1 positioning, though its effects were weaker compared to MinD2. This study provides the first direct evidence of MinD proteins regulating the positioning of tubulin superfamily proteins in archaea. In a complementary study, Patro et al. identified MinD2 as a determinant of motility and cell shape, influencing the positioning of chemosensory arrays and archaellum motors. Additionally, they found that MinD2 and MinD4 function synergistically to ensure proper cellular organization, highlighting the complexity of MinD-mediated processes in haloarchaea. Together, these two studies underscore the intricate regulation of motility and spatial organization in haloarchaea, improving our understanding on the functional diversity of this large family of ATPases in bacteria and archaea.

## Protease regulation

Intramembrane proteases play critical roles in cellular regulation, but their functions in archaea remain poorly understood. Costa et al. investigated the role of rhomboid proteases in *H. volcanii*, focusing on two homologs, rho1 (HVO_1474) and rho2 (HVO_0727), which were not essential for *H. volcanii* viability but significantly influenced motility and biofilm formation. The Δ*rho1* mutant exhibited increased motility and biofilm formation, reduced adhesion to glass surfaces, with profound morphological changes. In contrast, the double mutant (Δ*rho1* Δ*rho2*) showed enhanced adhesion, a mild reduction in motility (similar to Δ*rho2*), and fewer morphological abnormalities. Complementation studies revealed the function of the two proteases only partially overlapped, providing new perspectives on proteolytic regulation of biofilm formation and cell shape in archaea.

## Conclusion

These nine manuscripts significantly advance our understanding of the *Molecular, cellular, and ecological processes of haloarchaea*. As research in this field continues to evolve, these studies contribute to a strong foundation for future discoveries, and highlight the importance of haloarchaea as model organisms in archaeal research. We extend our sincere appreciation to all contributing authors, reviewers, and editorial board members for their efforts in assembling this compelling body of Research Topic. We hope that this Research Topic will serve as a valuable resource for researchers in microbiology, molecular biology, and applied biotechnology.

